# Open mesh plug repair for inguinal hernia after femoro-femoral arterial bypass: Two retrospective case series

**DOI:** 10.1016/j.ijscr.2019.08.017

**Published:** 2019-08-20

**Authors:** Shinji Onda, Katsuhito Suwa, Kai Neki, Katsuhiko Yanaga

**Affiliations:** Department of Surgery, The Jikei University School of Medicine, 3-25-8, Nishishimbashi, Minato-ku, Tokyo, 105-8461, Japan

**Keywords:** Inguinal hernia, Femoro-femoral arterial bypass, Mesh-plug repair

## Abstract

**Introduction:**

Reports on inguinal hernia repair after femoral arterial bypass are limited, and a recommended procedure has not been established.

**Presentation of case:**

Case 1. A 77-year-old man who had a history of femoro-femoral arterial bypass (FFB) for limb graft occlusion following endovascular aortic repair for abdominal aortic aneurysm presented with a left inguinal hernia. CT revealed an inguinal hernia and the FFB graft was identified in the subcutaneous plane. We selected mesh-plug repair under local infiltration anesthesia and his postoperative course was uneventful. Case 2. A 73-year-old man who had a history of FFB for occlusion the branch of the graft of endovascular stent for abdominal aortic aneurysm presented with a left inguinal hernia. CT revealed an inguinal hernia and the FFB graft was identified in the subcutaneous plane. We performed mesh-plug repair under general anesthesia and his postoperative course was uneventful. The patients are free of recurrence of the hernia or complication of the FFB graft as of 13 months and 30 months after the surgery, respectively.

**Discussion:**

We herein report two cases of successful open mesh plug repair for inguinal hernia after FFB.

**Conclusion:**

The mesh plug repair is safe and useful for the treatment of inguinal hernia after FFB, for which preoperative CT is helpful for understanding precise anatomy which facilitates surgical planning.

## Highlights

• We experienced two case of inguinal hernia after femoro-femoral arterial bypass (FFB).
• The mesh plug repair is safe and useful for the treatment of inguinal hernia after FFB.
• Preoperative CT is helpful for understanding precise anatomy which facilitates surgical planning.

## 1 Introduction

Inguinal hernia is a common condition and its repair is one of the most commonly performed surgical procedures. Currently, various procedures such as the Lichtenstein, mesh-plug, Kugel and laparoscopic repair have been developed and their usefulness has been widely reported [[1], [2], [3], [4]]. Inguinal hernia surgery after prosthetic arterial bypass is difficult because of the preperitoneal adhesion due to previous surgery and arterial bypass being an obstacle in the surgical field. To the best of our knowledge, reports on inguinal hernia repair after femoral arterial bypass are limited [[5], [6], [7]], and a recommended procedure has not been established. We herein report successful inguinal hernioplasty by anterior approach in two cases who had previous FFB. The research work has been reported in line with the PROCESS criteria [8].

## 2 Presentation of case

### 2.1 Case 1

**Fig. 1:**
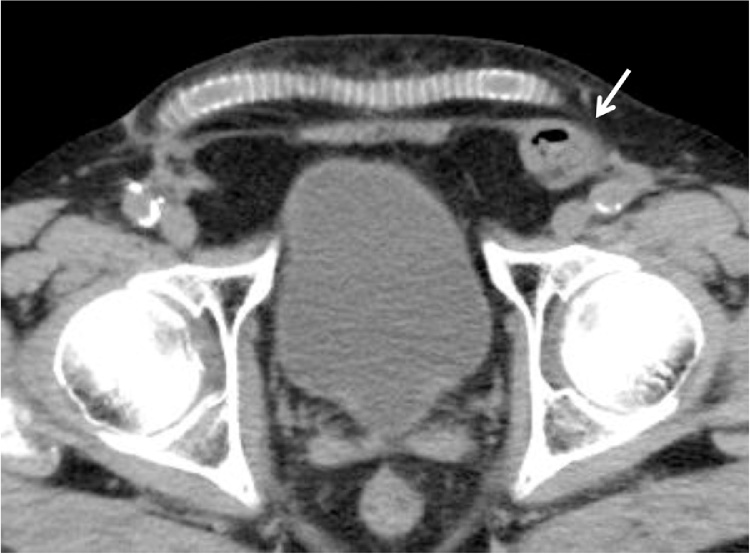
Case 1. Computed tomography revealed a left inguinal hernia (arrow) and femoro-femoral arterial bypass graft in the subcutaneous plane.

**Fig. 2:**
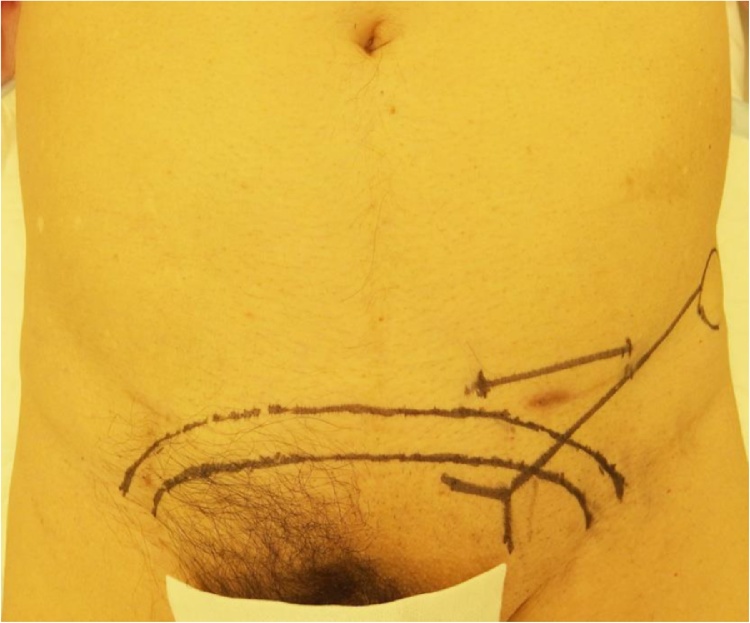
Case 1. The location of the subcutaneous femoro-femoral arterial bypass graft and a planned incision line were marked on the skin.

**Fig. 3:**
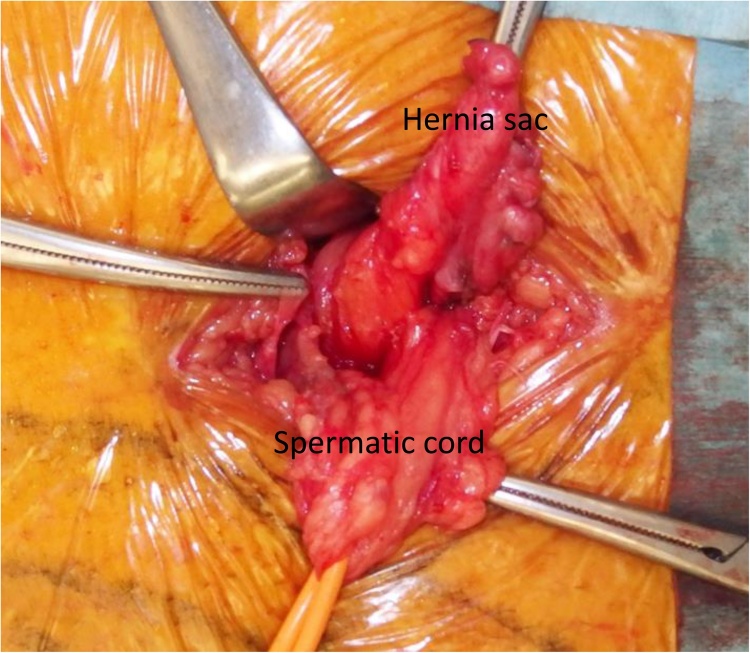
Case 1. An indirect hernia sac was identified, which was freed from the spermatic cord to the deep inguinal ring.

A 77-year-old man with left groin pain and swelling was referred to our hospital. He had a history of hypertension, dyslipidemia, interstitial pneumonia, chronic kidney disease, and myocardial infarction requiring percutaneous coronary interventions. He underwent aortic valve replacement and the Maze procedure for aortic valve regurgitation and atrial fibrillation at 68-year-old, followed 3 months later by endovascular aortic repair (EVAR) for abdominal aortic aneurysm. At one month after EVAR, he underwent femoro-femoral arterial bypass (FFB) for limb graft occlusion following EVAR. He also had a history of repeated ventricular tachycardia and underwent implantation of an implantable cardioverter defibrillator (ICD). In addition, he had prostate carcinoma treated with radiation therapy. On physical examination, the subcutaneous FFB graft was palpable in the suprapubic region, and left inguinal hernia was identified. Computed tomography (CT) revealed a left inguinal hernia and a FFB graft anastomosed between bilateral common femoral artery in the subcutaneous space anterior to the pubis (Fig. 1). Because the patient was considered at high risk for general anesthesia and had previous radiation therapy to the pelvis which can develop adhesions in the preperitoneal and peritoneal cavity, we scheduled an anterior tension-free hernioplasty under local infiltration anesthesia. Aspirin was continued in the perioperative period to reduce the risk of cardiovascular ischemic events. The location of the subcutaneous FFB graft was marked on the skin with a pen (Fig. 2). The surgical field was prepared and draped with an iodine-impregnated occlusive dressing, then step-by step local infiltration anesthesia was administered, starting prior to making the incision with the combination of 1% lidocaine with epinephrine 1:100,000 and 0.25% bupivacaine. A 6-cm skin incision was made from 1-cm above the center of the left inguinal ligament toward the midline, and the subcutaneous tissue was dissected carefully to avoid exposure of the FFB graft. The external oblique aponeurosis was opened and the spermatic cord was isolated, in which mild adhesion was observed. The cremasteric sheath was incised longitudinally and the internal ring was explored. An indirect hernia sac was identified, which was freed from the spermatic cord to the deep inguinal ring and inverted into the preperitoneal space (Fig. 3). Thereafter, a mesh-plug repair using lightweight mesh was subsequently performed. The operating time was 85 min. The FFB graft was not exposed or injured during the operation. The postoperative course was uneventful, and the patient was discharged on postoperative day 2. There has been no signs of recurrence of the hernia or the FFB graft infection, but he died from an aortic dissection 13 months after the hernioplasty.

### 2.2 Case 2

**Fig. 4:**
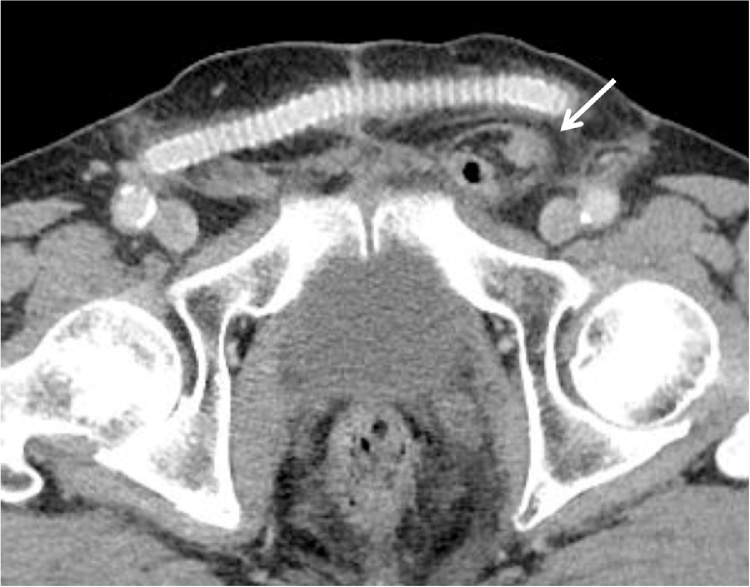
Case 2. Computed tomography revealed a left inguinal hernia (arrow) and femoro-femoral arterial bypass graft in the subcutaneous plane.

A 73-year-old man was referred to our hospital with left inguinal pain and swelling. He had a history of hypertension and dyslipidemia. He underwent endovascular stent graft replacement for abdominal aortic aneurysm at the age of 68 years. Four years after the operation, a branch of the graft was found to be occluded, for which he underwent FFB. He also had undergone surgery for lung cancer and radical prostatectomy for prostate cancer. CT revealed a left inguinal hernia and a FFB graft anastomosed between bilateral common femoral artery in the subcutaneous space anterior to the pubis (Fig. 4). Aspirin was discontinued 7 days before surgery. Because the patient had a previous radical prostatectomy without cardiopulmonary dysfunction, we selected mesh-plug repair under general anesthesia. The marking of the location of the FFB and surgical field preparation were performed in the same fashion as in Case 1. During the operation, a left indirect inguinal hernia was found for which a mesh-plug repair using lightweight mesh was performed. The operating time was 70 min. The FFB graft was not exposed or injured during the operation. His postoperative course was uneventful, and he was discharged on postoperative day 1. As of 13 months after the hernioplasty he remains well and without any recurrence of the hernia or FFB graft complications.

## 3 Discussion

We successfully performed hernioplasty by open mesh plug repair for inguinal hernia after femoro-femoral arterial bypass (FFB).

Inguinal hernia is widely recognized as a complication after radical prostatectomy [9,10]. Inguinal hernia repair after prostatectomy and radiation therapy to the lower abdomen are difficult condition known to complicate inguinal hernioplasty because of adhesion in the preperitoneal cavity. Furthermore, inguinal hernia repair after previous arterial grafting in the groin space such as femoro-femoral, aorto-femoral, axillary-femoral and ilio-femoral artery bypass has a risk of graft complications, including graft injury, infection and occluding.

Inguinal hernia and peripheral arterial disease which require arterial bypass surgery are both common in elderly people, and inguinal hernia repair after previous arterial bypass surgery will be performed more frequently in the future. Therefore, a management strategy for this condition should be developed.

Differentiation of inguinal hernias and femoral hernias is often difficult by clinical examination alone. CT is valuable for confirmation of the diagnosis of inguinal hernia [11]. In our patients, preoperative CT provided precise anatomical information of the inguinal hernia and arterial grafting which facilitated surgical planning. The FFB is placed in prefascial subcutaneous plane in most patients, but some cases in a preperitoneal position according to the variable conditions of the abdominal wall such as prior surgery, some skin changes, and thin subcutaneous fat layer.

According to our knowledge, only three case reports on inguinal hernia after femoral arterial bypass surgery have been reported, in which CT was performed in two cases [6,7].

We usually perform Lichtenstein or mesh-plug repair for patients with inguinal hernia after previous surgery or radiation for preperitoneal cavity. Thus, we selected mesh-plug repair for these patients.

Radical prostatectomy leads to an increased risk of inguinal hernia development [12], but the etiology is still not fully understood. Regarding the type of inguinal hernia after radical prostatectomy, the incidence of indirect type was reported to be 91–100%, which is much higher than that of direct type [[13], [14], [15], [16], [17]]. Because the Case 2 had also undergone radical prostatectomy, mesh-plug repair was considered to be appropriate.

To our knowledge, three cases of laparoscopic hernia repair after femoral artery bypass have been reported [[5], [6], [7]]. However, in the cases presented herein, laparoscopic hernia repair was judged technically difficult and could have increased the risk of arterial graft complication. Dissection of preperitoneal cavity with firm adhesion and mesh placement is difficult, which results in insufficient reinforcement and may injure the arterial graft during mesh fixation. There was a case report of hybrid laparoscopic and anterior approach for postsurgical inguinal hernia after iliofemoral arterial bypass [6]. In this report, initially a laparoscopic approach was performed, but the mesh fixation was insufficient because dissection of the preperitoneal space was insufficient and the margin for the hernia orifice was minimal, which required an anterior mesh replacement using the Lichtenstein method.

In addition, laparoscopic hernioplasty requires general anesthesia, but in high risk patients with significant cardiopulmonary disease, general anesthesia may be at increased risk for postoperative cardiopulmonary complications. On the other hand, anterior repair such as Lichtenstein’s method or mesh-plug repair for inguinal hernia can be safely performed under local infiltration anesthesia.

Regarding surgical technique, the surgical field should be exposed with gentle retraction and maintained by pulling the tissue with attached subcutaneous fat to avoid exposure and injury of the arterial grafting. Excessive retraction and extensive dissection may increase the risk of graft infection, injury and occlusion.

## 4 Conclusion

The optimal technique for repair of inguinal hernia after FFB seems to be the mesh plug repair or Lichtenstein’s method. Preoperative CT is helpful for understanding precise anatomy of the arterial grafting which facilitates surgical planning.

## Funding

There are no sources of funding for this research.

## Ethical approval

This study has been exempted by our institution.

## Consent

Consent has been obtained from the patients. No identifying details have been used in the article.

## Author contribution

S ONDA: study concept and draft the article. K NEKI: collected the data and treated the patients. K SUWA: discussed the content of the manuscript and revised the article. K YANAGA: edited the article. All authors read and approved the final manuscript.

## Registration of research studies

Researchregistry4934.

## Guarantor

Shinji Onda.

## Provenance and peer review

Not commissioned, externally peer-reiewed.

## Declaration of Competing Interest

No potential conflict of interest relevant to this article was reported.
